# Epithelial-to-Mesenchymal Transition and MicroRNAs in Lung Cancer

**DOI:** 10.3390/cancers9080101

**Published:** 2017-08-03

**Authors:** Antoine Legras, Nicolas Pécuchet, Sandrine Imbeaud, Karine Pallier, Audrey Didelot, Hélène Roussel, Laure Gibault, Elizabeth Fabre, Françoise Le Pimpec-Barthes, Pierre Laurent-Puig, Hélène Blons

**Affiliations:** 1 INSERM UMR-S1147, CNRS SNC 5014, Saints-Pères Research Center, 45 rue des Saints-Pères Paris-Descartes University, Sorbonne Paris Cité University, 75006 Paris, France; antlegras@gmail.com (A.L.); nicolas.pecuchet@me.com (N.P.); pallier.karine@gmail.com (K.P.); audrey.didelot@parisdescartes.fr (A.D.); elizabeth.fabre@aphp.fr (E.F.); pierre.laurent-puig@parisdescartes.fr (P.L.-P.); 2 Thoracic Surgery and Lung Transplantation Department, Georges Pompidou European Hospital, 20 rue Leblanc, Assistance Publique-Hôpitaux de Paris, 75015 Paris, France; francoise.lepimpec-barthes@aphp.fr; 3 Medical Thoracic Oncology Department, Georges Pompidou European Hospital, 20 rue Leblanc, Assistance Publique-Hôpitaux de Paris, 75015 Paris, France; 4 INSERM UMR-S1162, 27 rue Juliette Dodu, 75010 Paris, France; sandrine.imbeaud@inserm.fr; 5 Pathology Department, Georges Pompidou European Hospital, 20 rue Leblanc, Assistance Publique-Hôpitaux de Paris, 75015 Paris, France; helene.roussel@aphp.fr (H.R.); laure.gibault@aphp.fr (L.G.); 6 INSERM UMR-S970, Paris Centre de Recherche Cardiovasculaire, Georges Pompidou European Hospital, 20 rue Leblanc, 75015 Paris, France; 7 Molecular Biology Department, Georges Pompidou European Hospital, 20 rue Leblanc, Assistance Publique-Hôpitaux de Paris, 75015 Paris, France

**Keywords:** epithelial-mesenchymal transition, microRNAs, lung neoplasms, biomarkers, tumour

## Abstract

Despite major advances, non-small cell lung cancer (NSCLC) remains the major cause of cancer-related death in developed countries. Metastasis and drug resistance are the main factors contributing to relapse and death. Epithelial-to-mesenchymal transition (EMT) is a complex molecular and cellular process involved in tissue remodelling that was extensively studied as an actor of tumour progression, metastasis and drug resistance in many cancer types and in lung cancers. Here we described with an emphasis on NSCLC how the changes in signalling pathways, transcription factors expression or microRNAs that occur in cancer promote EMT. Understanding the biology of EMT will help to define reversing process and treatment strategies. We will see that this complex mechanism is related to inflammation, cell mobility and stem cell features and that it is a dynamic process. The existence of intermediate phenotypes and tumour heterogeneity may be debated in the literature concerning EMT markers, EMT signatures and clinical consequences in NSCLC. However, given the role of EMT in metastasis and in drug resistance the development of EMT inhibitors is an interesting approach to counteract tumour progression and drug resistance. This review describes EMT involvement in cancer with an emphasis on NSCLC and microRNA regulation.

## 1. Introduction

Epithelial-to-mesenchymal transition (EMT) is an evolutionarily conserved but complex molecular and cellular program in which cells undergo conversion from epithelial to mesenchymal state [1]. Epithelial differentiated characteristics are lost, including cell-cell adhesion, planar and apical-basal polarity and lack of mobility. On the contrary, mesenchymal features are briefly acquired, such as cell mobility, invasiveness, gain of stem cell properties and a reinforced resistance to apoptosis. EMT was initially described as a cell culture phenomenon before being recognized in vivo and studied in embryonic development. This process is obviously reversible and is highly conserved from diploblasts (medusa) 800 million years ago to nowadays [2]. It aims at dissociating epithelium and degrading basement membranes so that cells acquire a fibroblastic or glial-cell phenotype and finally migrate. This state was defined as “mesenchyme” (from Greek, μεσο, μεσο, middle and ενκηυμα in fusion) to describe a poorly organized state between two tissues. After migration, such cells are able to reverse their phenotype with the mesenchymal to epithelial transition (MET). EMT and MET are included under the term epithelial-mesenchymal plasticity [3]. Both play an important play in organogenesis (i.e., in renal epithelium or cardiac organogenesis formation) [4] but also in cancer [5,6].

### 1.1. Cellular Pathways and EMT

Many signalling pathways control EMT according to the different cellular contexts [5,7]. Transforming Growth Factor β TGFβ) [8] and Epidermal Growth Factor (EGF) [9] pathways have been extensively studied but others are also known to drive EMT in specific situations (tumour cells including NSCLC): the Fibroblast Growth Factor (FGF) [10,11], Hepatocyte Growth Factor (HGF) [12], Platelet-derived Growth Factor (PDGF), Insulin-like Growth Factor (IGF) [13], Vascular Endothelial Growth Factor (VEGF), Oestrogens, Hypoxia [14,15], Autocrine Motility Growth Factor (AMF), bile acids, nicotine, ultraviolet light, integrins, Wnt, Notch [16], Interleukin-related Protein (ILEI), Interleukin-6 (IL-6), Sonic hedgehog (Shh), Bone Morphogenetic Protein (BMP), Stem Cell Factor (SCF), cyclooxygenase-2/prostaglandin E2 (COX-2/PGE2) [17] and also extracellular matrix changes, as shown for collagen I and hyaluronan [18,19]. Depending on the experimental model and the stimulation nearly all signalling pathways may eventually promote a mesenchymal transition.

### 1.2. Transcriptional Regulation of EMT

Several transcription factors act as molecular switches for the EMT program [20]. The loss of E-cadherin is a hallmark of EMT. This cadherin is responsible for cell cell adhesion and cytoskeleton organisation. Its loss leads to the conversion from epithelial cells to motile and invasive mesenchymal cells [21] which is orchestrated by transcriptional repressors [5]. Direct transcriptional repression of E-cadherin is coordinated by several transcription factors interacting together. SNAIL superfamily members (such as zinc finger proteins SNAI1/SNAIL and SNAI2/SLUG) interact with several co-repressors and epigenetic remodelling complexes to repress E-cadherin through N-terminal SNAG domain binding to the E-box promoter sequence [22]. Other direct repressors are ZEB family members ZEB1 (Zinc finger E-box-binding homeobox 1, previously known as TCF8 and δEF1) and ZEB2/SIP1, E47 and the Krüpple-like factor 8 (KLF8) [23,24]. Furthermore, TWIST family members (basic helix-loop-helix transcription factors TWIST1 and TWIST2), Goosecoid, E2.2/TCF4 and FOXC2 indirectly repress E-cadherin transcription [6]. TWIST is a transcription factor fully implicated in EMT [25]. Its proteins belong to the family of basic helix-loop-helix transcription factors which are able to modulate expression of different target genes through E-box responsive elements [26]. TWIST1 and TWIST2, highly conserved, share the Twist-box protein interaction surface in their C-terminal half [27]. TWIST forms functional homo- and heterodimers with TCF3/E2A and Hand2, the balance between homo- and heterodimers regulating limb development and cranial suture fusion. In adulthood, *TWIST1* expression was reported in mesoderm-derived tissues including heart, skeletal muscles, placenta [27] and also brown fat with a specific thermoregulation function [28]. TWIST proteins physically interact with NF-κB and specifically prevent its ability to activate pro-inflammatory cytokine-encoding genes [29]. Furthermore, TWIST proteins reduce TNFα induction response to T-cell receptor activation [30].

EMT network is highly controlled. The high-mobility group protein HMGA2 and the homeodomain-containing protein SIX1 act as a coordinator of EMT inducers [31,32]. In development and in carcinogenesis, SNAIL1 appears at the onset of EMT then SNAIL2, ZEB, E47 and TWIST are induced to maintain the migratory mesenchymal state [33] suggesting an orchestration in time of the process. Moreover EMT transcription factors may undergo post-translational regulation. For example SNAIL1 nuclear stabilization is promoted by NF-κB [34] and the zinc transporter LIV1 [35]. 

### 1.3. Junctions, Cytoskeleton and Matrix

The cadherin switch through the loss of E-cadherin represents a universal marker of EMT. E-cadherin is the major constituent of epithelial adherens junctions which mediate intercellular adhesion with tight junctions, forming the zonula adherens. Epithelial cells loose cell-cell adhesion and cell polarity. Following E-cadherin loss, the expression of mesenchymal markers such as vimentin, fibronectin, N-cadherin, alpha-smooth muscle actin (αSMA), and the activity of matrix metalloproteinases (MMP-2, MMP-3, MMP-9) increase. SNAIL1 represses the expression of tight junction components such as claudin-3, -4 and -7 [36]. Furthermore, EMT inducers directly repress the protein complexes involved in apico-basal polarity of epithelial cells (Par, Crumbs and Scribble) [37] and enhance expression of metalloproteinases that degrade the basement membrane, thereby favouring cell migration and invasion. Interestingly, MMP3 can trigger EMT through a positive regulatory feedback loop [38]. Finally, EMT transcription factors induce the expression of mesenchymal proteins such as fibronectin and N-cadherin [39] and are involved in the remodelling of the actin cytoskeleton [21]. The complexity of interactive downstream effector pathways and the fact that EMT is not a simple matter of changes in cell adhesive capabilities or in cytoskeletal organization leads to a wide range of different profiles of expression of the markers described [21]. The choice of the markers and the changes in their expression levels will depend upon the diversity of signals inducing EMT.

## 2. EMT in Cancer

### 2.1. Carcinogenesis

Recent evidences show that transcription factors linked to EMT may directly be involved in carcinogenesis. TWIST and ZEB proteins can prevent cells from undergoing oncogene-induced senescence and apoptosis by inhibiting both TP53 and RB-dependent pathways, leading to a deregulation of MYC, RAS, ERBB2 and cell cycle inhibitors P14, P16 and P21 expression [40,41]. This may explain how TWIST1 and TWIST2 can cooperate with an activated version of RAS to transform mouse embryonic fibroblasts [41]. Furthermore, TWIST proteins also downregulate PP2A phosphatase activity and efficiently cooperate with an oncogenic version of H-RAS in malignant transformation of human mammary epithelial cells, leading to claudin-low tumours, which are believed to be the most primitive breast malignancies [42]. Thus, by downregulating crucial tumour suppressor functions, EMT inducers make cells particularly prone to malignant conversion. Beside this effect, EMT was linked to progression and metastasis through its effects on matrix, motility and gain of stem cell properties.

Both *TWIST* genes were reported overexpressed in many types of cancers: prostate, breast, cervical, endometrial, ovarian, head and neck cancer, oesophageal squamous cell carcinoma, gastric, hepatocellular carcinoma, pancreas, colon, kidney, glioma, melanoma, neuroblastoma, parathyroid, pheochromocytoma, sarcoma [41,43] and also NSCLC [44].

TWIST proteins are reported to induce EMT and to be one of its main actor: TWIST induces loss of E-cadherin-mediated cell-cell adhesion and an EMT in epithelial cells [45,46]. In NSCLC, the overexpression rate of TWIST proteins was 38% in tissues samples and results correlated with mRNA high level and N-cadherin expression [47]. Such a pattern was associated with worst prognosis. In cell lines, TWIST down expression inhibited cell invasion and increased apoptosis [47]. In *Epidermal Growth Factor Receptor* (*EGFR*)-mutated lung adenocarcinoma, we previously reported on surgical samples that TWIST1 expression was linked to *EGFR* mutations, low E-cadherin expression and low disease-free survival [44]. In cell lines, we demonstrated that EMT and the associated cell mobility were dependent upon TWIST1 expression in cells with *EGFR* mutation. Moreover a decrease of EGFR pathway stimulation through EGF retrieval or an inhibition of TWIST1 expression by small RNA technology reversed the phenomenon [44]. Such results are consistent with with EGF promoting E-cadherin endocytosis to induce EMT [48] but also induction of both SNAIL and TWIST [9,49]. EMT is common in NSCLC [50] and could in some patients be related to tobacco exposure. Indeed, cigarette smoke was proved to induce EMT [51,52]. In lung adenocarcinoma cell lines (H358), cigarette smoke extracts was able to induce EMT with activation of SRC kinase and the SRC kinase inhibitor PP2 inhibited cigarette smoke-stimulated EMT changes, suggesting that SRC is critical in cigarette smoke-stimulated EMT induction [53].

### 2.2. Lymph Node Metastases

To the best of our knowledge, presence of EMT-MET phenomenon remains poorly studied in the metastatic lymph nodes. In gastric cancer, the expression of N-cadherin in metastatic lymph nodes was associated to a bad prognosis [54]. An other study related the heterogeneity between primary tumours and metastatic lymph nodes in oesophageal cancer, with distinct EMT phenotypes and thus, a novel independent prognostic indicator [55]. Similar results were found in head and neck cancer [56] and breast cancer [57]. Concerning NSCLC, only one study investigated EMT markers in metastatic lymph nodes [58]. Expression of Brachyury in 115 surgically resected primary NSCLC and the corresponding metastatic lymph node samples were evaluated by immunohistochemical staining. Brachyury is a highly conserved cellular protein that belongs to the T-box transcription factor family and was reported to be essential for mesoderm formation in the early embryo [59]. In recent studies Brachyury was also linked to the EMT process during cancer progression [60]. Gene expression was associated with IL-8 expression and inversely associated with E-cadherin expression in NSCLC [14]. In metastatic lymph nodes Brachyury expression was significantly higher than in the primary tumour and lymph node level of expression was inversely associated with survival [58]. In models of NSCLC, lymphangiogenesis leads to proliferation, invasiveness and nodal metastasis [61]. Vascular endothelial growth factors (VEGF) -C and -D and their corresponding receptor (VEGFR3/Flt4) are the main actors in the development of tumour-associated lymphatic vessels [62]. They recruit endothelial cells and others stromal cells to develop and maintain an unrefined lymphatic network within the tumour microenvironment [62,63]. Furthermore, they might be involved in pre-metastatic lymph nodes by preparing the lymphatic vasculature to host the cancer cells [64]. These markers were correlated in NSCLC to nodal metastasis and, thus, patient survival, with heterogeneous results [65]. A meta-analysis found a correlation between VEGF-C, Lymphatic Vessel Density and lymph node metastasis in NSCLC [65]. Links between lymphangiogenesis and EMT remain to be elucidated but EMT markers were already associated with lymphatic vessel density in surgical specimen of NSCLC [66].

### 2.3. Distant Metastases

Development of metastasis involves distinct steps with specific underlying molecular mechanisms, which are: detachment of tumour cells from the primary tumour, invasion into surrounding tissues, intravasation into blood or lymphatic vessels, dissemination in the blood stream or the lymphatic system and finally, extravasation and outgrowth at a secondary site [21]. Despite solid evidence showing EMT implication in distant metastases [67], the metastatic process remains unclear for many points. Reversion of EMT (MET) seems to play a role in the metastatic process, MET could at some points explain the histologic similarity between metastases and primary tumours. First, different observations reported association of MET with metastatic tumour formation: (i) epithelial phenotype seems to be of importance in the formation of secondary tumours, as epithelial characteristics were highly associated with increased distant colonization after blood stream injections [68]. (ii) E-cadherin positive metastatic foci were found after injections of mesenchymal-like breast cancer cells, presenting a direct demonstration of need for E-cadherin expression [69]. Escape from primary tumour, gain of cell mobility and invasion may at first step be EMT-dependent, then cancer cells should undergo MET in the secondary organ. The regulation of phenotype plasticity remains largely unknown but it can be hypothesized that while the tumour microenvironment promote the induction and continuation of EMT, circulating tumour cells revert to an epithelial state due to the loss of EMT-inducing signal, before entering into metastatic sites [70]. Nevertheless, Aokage and colleagues [71] showed that MET appeared after the tumour cells arrival at the metastatic site, and considered that local microenvironment or local resident cells contribute largely to the MET. Different histopathological analyses suggested very close contact between metastatic carcinoma cells and the neighbouring parenchyma cells, supporting a possible E-cadherin-dependent linkage [72,73,74]. This ability to create heterotypic E-cadherin adhesions and related survival signals is coherent with the dormancy of the tumour cells and its low metabolism inherent to this micrometastatic stage [75]. 

Nevertheless, there are some debates about the implication of EMT in cancer progression [76,77] as aggressive tumours may, in some cases, develop metastases in the absence of EMT-MET. A point-counterpoint review, in 2005, proclaimed that the biological machinery of normal and malignant cells is sufficient to account for the events and processes observed without needing to objective radical change in cell phenotype like EMT [78] suggesting that EMT is not always a prerequisite to metastasis and that tumours may undergo partial or incomplete EMT [68]. Indeed, mesenchymal cells derived from epithelial tumour cells are very difficult to distinguish from stromal cells or other tumour-associated fibroblast. However, EMT was described at the invasive front of tumour as small aggregates of tumour cells extending from the tumour mass into adjacent stroma [79]. Cancer cells have a broad repertoire of invasion mechanisms, and cell-type-specific patterns of cell migration can be classified into single cell migration (amoeboid, mesenchymal) and collective migration modes (cell sheets, strands, tubes, clusters) [80]. The collective migrating cells form membrane protrusions, such as ruffles and pseudopods, use cell-matrix adhesion receptors and, in contrast to solitary migration, do not retract their cellular tails but rather exert pulling forces on adjacent adherent cells [81,82]. An other potential mechanism for tumour cell invasion and metastasis is the podoplanin-mediated remodelling of actin cytoskeleton and tumour invasion [83], also described in NSCLC [84].

## 3. EMT-Related MicroRNAs

MicroRNAs (miRNA) are highly conserved small single-stranded non-coding RNA of 21-23 nucleotides acting as post-transcriptional regulators of gene expression. They function to affect RNA stability and translation in order to negatively regulate gene expression [85]. MicroRNAs are transcribed from DNA by RNA polymerase II or III as a form of long primary transcripts (pri-miRNA) [86] which are then processed by the microprocessor complex containing RNase III enzyme Drosha and DGCR8 (DiGeorge syndrome chromosomal region 8) into shorter stem-loop-structured double-stranded RNA (hairpin precursor miR, pre-miR) [87]. Pre-miR are delocalized from nucleus to cytoplasm and processed into mature-miR by RNase enzyme III Dicer [88]. The RNA interference is finally efficient into the RNA-induced silencing complex (RISC) to target single-stranded complementary messenger-RNA (mRNA) for translation repression or mRNA degradation, by binding to the 3’ untranslated regions of their target genes [89]. The target mRNA could be blocked in case of partial complementarity or degraded in case of perfect complementarity [90]. Thus, the possible imperfect match offers the ability to regulate many genes.

In normal lung, the overall expression profile of miRNAs is 75% similar in mouse and human lung, indicating evolutionary conservation of miRNAs expression [91]. However, miRNAs expression profile varies along development and profiles differ between human foetal, post-natal and adult lung [91]. In adult lung tissues the 30 most highly expressed miRNAs were identified as [91]: miR-103, miR-99a, miR-15b, miR-150, miR-320, miR-23a, miR-200c, miR-195, miR-27b, miR-199s, miR-92, miR-29b, miR-30d, let-7g, miR-223, miR-199a, miR-30c, miR-142-3p, miR-125a, miR-26b, miR-29a, miR-126, miR-29c, miR-16, let-7b, miR-145, miR-21, let-7a, miR-30b and miR-26a. In parallel, tissue-specific miR were found without any correspondence with the previous list [92]: miR-224, miR-137, miR-192, miR-886, miR-31, miR-92b, miR-10a, miR-625, miR-301a, miR-96 and let-7i.

Reports of miRNA implication in diseases began in the 2000s, with a first description that miRNAs were dysregulated in human B-cell chronic lymphocytic leukemia using a microarray containing miRNA probes [93]. MicroRNAs were then studied in many situations, hepatic viral infections [94], Alzheimer disease [95], cardiac hypertrophy [96], diabetes [97] and in various lung diseases such as chronic obstructive pulmonary disease, sarcoidosis, pulmonary fibrosis [98,99]. MicroRNAs have been shown to play a crucial part in cancer development and progression in the past decade, in various solid organ cancers. Half of miRNAs genes are located at fragile sites or genomic regions involved in cancer-related chromosomal abnormalities [100]. MicroRNAs were then characterized as “OncomiR” or “Tumour suppressor miR” depending on the suppressed target genes [101,102,103]. Finally, miRNAs functions studies showed their capacity to affect pathways regulating EMT [1,76,104]. The miRNAs regulatory network is highly complex and deeply integrated into cell functions. The different studies may be heterogeneous and difficult to compare, different models can be used including cancer tissues and cell lines. Moreover, despite tissue preservation, RNA studies remain shaky and submitted to numerous pitfalls before final interpretation. Two main technologies allow miRNAs analyses qPCR based and specific probes sets or high throughput sequencing. Concerning EMT, the main miRNAs involved are in one hand the miR-200 family and miR-205 that maintain the epithelial cell phenotype [104,105,106,107,108] and in the other hand, miR-21 is up-regulated in many cancers. It facilitates TGF-β-induced EMT [8] and was the first OncomiR to be identified. Based on comparison between tumour and normal tissue levels of expression, approximately 200 miRNAs were shown dysregulated in NSCLC [103]. MicroRNAs from the miR-200 family (miR-200a/200b/200c/141/429) have been shown to inhibit EMT, cell migration and invasion by targeting ZEB1 and ZEB2 mRNA, two repressors of E-cadherin expression [106]. Based on their chromosomal location, the miR-200 family can be split into two different gene clusters: miR-200/200b/429 (chromosome 1) and miR-200c/141 (chromosome 12) [106]. Loss of these genomic loci and subsequently loss of miRNAs expression was reported in mesenchymal cancer cell lines and linked to cancer progression including NSCLC [107]. Down-regulation of miR-200 family was also documented in cases with hypermethylation of the DNA locus [109,110]. Inversely, miR-200c has been shown to inhibit the metastasis in A549 NSCLC cell lines [111,112]. Bracken et al. showed that the promoter for the pri-miR (shared by miR-200a, miR-200b, and miR-429) is located within a 300-bp segment located 4 kb upstream of miR-200b. This promoter region is sufficient to confer expression in epithelial cells and is repressed in mesenchymal cells by ZEB1 and SIP1 through their binding to a conserved pair of ZEB-type E-box elements, located proximal to the transcription start site [113,114]. Therefore depending on extra cellular stimulations the miR-200/ZEB1/2 equilibrium may turn on epithelial or mesenchymal markers. Moreover, several other miRNAs were associated with NSCLC progression as miR-224 [115] or miR-1247 [116]. 

Several studies have indicated that miRNAs frequently form feedback loops, since they are regulated by transcription factors which they directly or indirectly target [117]. Siemens et al. showed that miR-34a could downregulate SNAIL as well as SLUG and ZEB1. Conversely, SNAIL can repress miR-34a by binding to E-box sequences in the miR-34a promoter thereby forming a double negative feedback loop blocking cell in a mesenchymal state [117,118]. 

Furthermore, in parallel to EMT regulation, miRNAs play crucial roles in carcinogenesis. Examples are (i) the negative regulatory loop between NF-κB and miR-146. In the highly metastatic human breast cancer cell line MDA-MB-231, lentiviral-mediated expression of miR-146a/b significantly downregulated the IL-1 receptor-associated kinase and the TNF receptor-associated factor 6, two key scaffold proteins in the IL-1 and Toll-like receptor signalling pathway, known to positively regulate NF-κB activity [119]. (ii) The tumour suppressor miR-34a targets CDK4/6, MET, HDAC1, E2F3 and Bcl-2 and induces cell cycle arrest and apoptosis [117]. 

## 4. MicroRNAs, EMT and NSCLC

In NSCLC, probably due to tumour heterogeneity and to different technical and analytical issues, it is difficult to find a common signature of miRNAs expression. Wang et al. [103] reviewed 4 studies comparing miRNAs profile in NSCLC tissues versus the corresponding non-cancerous lung tissues and pointed out that miRNAs identified in each study are different from the others [120,121,122]. However Zadran et al. [123], described a cancer-specific miRNAs signature for different solid organ cancers including lung, and Vosa et al. [124] presented the 30 most differentially expressed miRNAs in NSCLC. Supplementary Table S1 shows different published miRNAs signatures in NSCLC [120,121,122,123,124,125]. More than 150 miRNAs were identified as markers of NSCLC form 6 large studies. MiR-210, miR-143 and miR-205 were recurrently linked to NSCLC in 3 or 4 out of 6 studies. Three miRNAs (miR-195, miR-224 and miR-124a-1) are either up or downregulated and finally 130/153 miRNAs were associated to NSCLC in only 1 out of 6 studies. This illustrates the difficulties of validating relevant markers in clinics, due to the absence of specific miRNAs signature in NSCLC. 

However miRNAs was evaluated as diagnostic tool, in sputum [126,127,128] or in plasma/serum [129] for the early detection of NSCLC. In plasma, or serum and/or exosome, different tools were elaborated, with various techniques reviewed by Hou et al. [130]. Several authors proposed single miRNA or multiple-miRNA panels useful for NSCLC screening. However, these studies remain to be validated given the heterogeneity of the normalization methods and the starting material used for RNA isolation [129,130,131,132,133].

**Figure 1:**
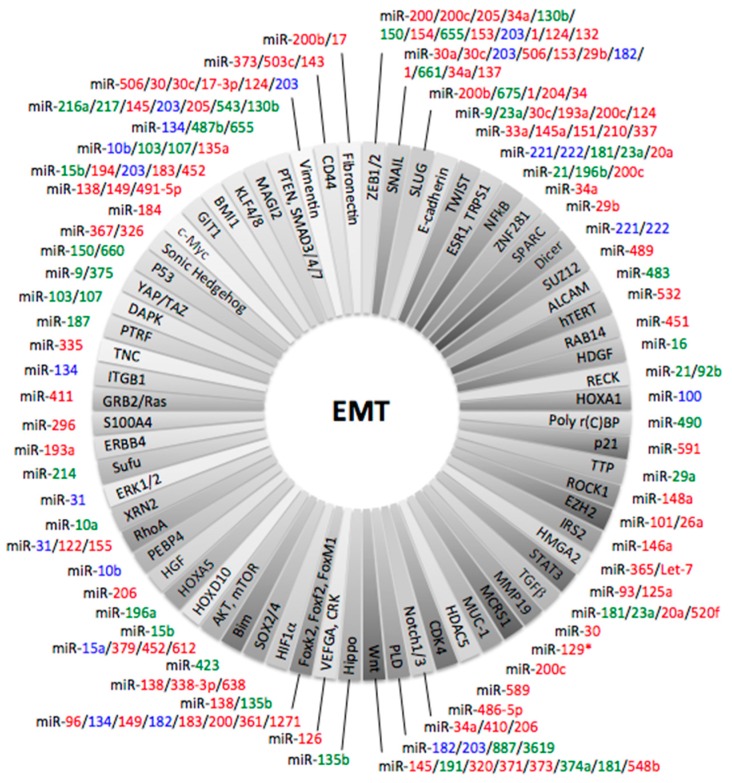
EMT regulation by miR in cancer, a complex network (Colour legend: green, promote EMT; red, suppress EMT; blue, controversial) (adapted from [117,205,206,207,208,209,210]).

To summarize published data concerning miRNAs and EMT in NSCLC, a systematic review of literature in Medline identified more than 75 articles based on cell lines studies (Table 1). Another subset of 37 articles was retained, based on studies on human NSCLC specimens leading to identify 35 miRNAs as modulator of EMT (Table 2). Figure 1 summarizes involved miRNAs in EMT in cancer. 

To go further in the interpretation of the data, regulation of TWIST1 by miRNAs was investigated by Nairismägi et al. [148]. They identified 18 miRNAs targeting *TWIST1*’s 3’UTR region but only 3 were able to significantly repress *TWIST1* translation: miR-145a-5p, miR-151-5p and in combination: miR-145a-5p + miR-151-5p and miR-151-5p + miR-337-3p. Among the many roles attributed to miRNAs in cancer, understanding their interaction with oncogenes and signalling pathways is of importance as it could lead to treatment strategies. In NSCLC, activation of the EGFR pathway plays a critical role in tumour development. A subset of miRNAs has been shown to interact with the EGFR pathway either as activators (miR-21, 24, 25 or miR-7) or repressors (miR-133) and may therefore modulate EGFR pathway activation. Concerning RAS activation and miRNAs in NSCLC, miR-31 was shown to target 6 regulators of the RAS/MAPK pathway and regulate lung epithelial cell growth. MiR-31 has been described to cooperate with RAS to drive lung tumorigenesis. Studies have revealed that miRNAs constitute a regulatory network in the post-transcriptional regulation of pathway genes. In parallel, tumour genetic background seems to impact on miRNAs profiles. Indeed, specific miRNAs profiles are associated to *EGFR*, *KRAS* or WT tumours [211]. In a high-throughput screening program in NSCLC, miR-155 was upregulated only in *EGFR/KRAS* negative group, miR-25 was upregulated only in *EGFR* positive group and miR-495 was upregulated only in KRAS positive adenocarcinomas [211].

We have previously reported an oncogenic cooperation between EGFR activation and TWIST1 reactivation in *EGFR* mutated NSCLC. Using NSCLC cell lines, Takeyama et al. showed that *EGFR* mutated cell lines had more epithelial characteristics as compared to non-*EGFR* mutated cell lines and that the mesenchymal state in the second group was related to ZEB1 upregulation [212]. It seems therefore that mutational status could drive specific EMT pathways. 

For other pathways involved in NSCLC, experiments suggested that miR-19 induces EMT [213]. MiR-19 and miR-21 modulate PTEN levels [213]. High miR-19 levels were found in NSCLC cells and experiments suggest that PTEN is involved in miR-19-induced EMT, migration and invasion [213].

Inflammatory cytokines and hypoxia have been proven to promote EMT. NF-κB itself is regulated by miRNAs through different regulatory loops of which the mir-146/NF-κB was documented in A549 cell line [214]. Moreover, NF-κB-mediated inflammation was shown to lead to EMT due to decrease in miR-200c [191] and in nuclear stabilization of SNAIL1 [34]. Hypoxia directly triggers EMT through the ubiquitin C-terminal hydrolase-L1 (UCH-L1) and HIF-1α deubiquitination [215]. *TWIST1* is a direct target of HIF1-α. In cancer cells, hypoxia was shown to reactivate TWIST1 [6], allowing cell to disseminate to a less hostile microenvironment [14,15]. Furthermore, hypoxia induces SNAIL in NSCLC [216]. Relations between ROS and EMT have been established [217,218]. ROS can activate NF-κB signalling and Wnt-β-catenin signalling pathway [217].

As seen, interaction loops between miRNAs and oncogenes or miRNAs and EMT transcription factors were largely described and seem cell and/or model dependent. It is sometimes hard to decipher whether miRNAs dysregulation is at the origin or is a consequence of the EMT process. Approximately half of miRNAs are associated with CpG islands [219] and several studies illustrated that methylation status could be responsible for the dysregulated expression of miRNAs in NSCLC [116,202,220,221]. Such a phenomenon was described for miR-200 inactivation under SNAIL1 control to maintain the mesenchymal phenotype [222]. In addition, histone acetylation may influence miRNAs expression [223]. Histone modifications were identified as the main mechanisms of miR-212 silencing in NSCLC [224]. DNA methylation and histone modifications are also largely associated with EMT regulation [225].

An emergent pathway in cancer is the involvement of Prion protein (PrP) [226]. Several studies reported overexpression of the PrP in cancer and particularly, links with EMT via direct influence of PrP on neural cell adhesion molecules (NCAM) [227], certain matrix metalloproteinases [228] and Fyn activation [229].

**Figure 2:**
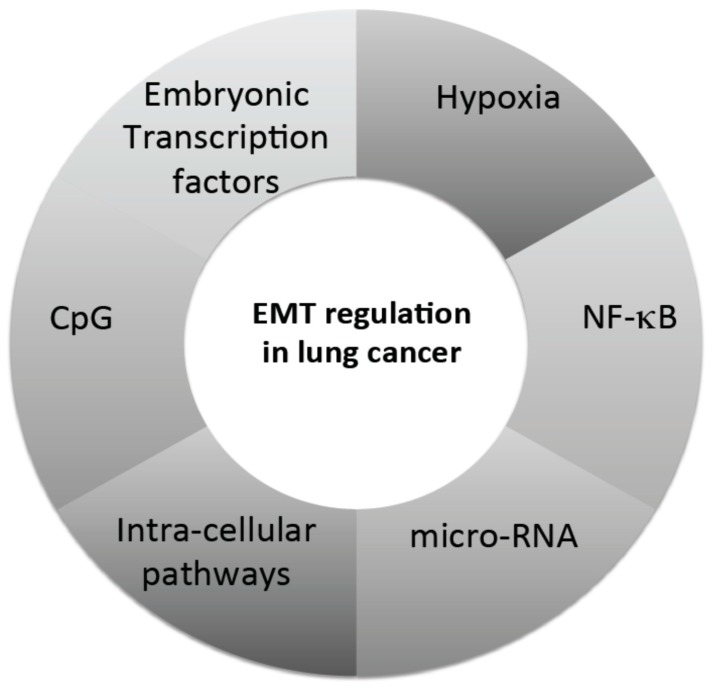
Hallmarks of the complex regulation of EMT in NSCLC. The regulation of EMT in NSCLC is based on several intricate conditions and actors, detailed in the Section 4 (adapted from [24,44,111,116,148,191,202,206,207,216,220,221]).

As a summary, Figure 2 illustrates the complex regulation of EMT in NSCLC.

## 5. Clinical Impact of EMT in NSCLC

EMT is largely involved in cancer development and in metastatic progression. For that it was frequently associated with prognosis [14,44,230,231,232]. The prognostic impact of miRNAs was investigated and some authors proposed prognostic miRNAs signature, in gastric and in NSCLC [233]. Using a cohort of 112 NSCLC patients, the last study identified a 5-miRNA-signature including 3 high-risk miRNAs (miR-137, miR-182*, and miR-372) and 2 protective miRNAs (miR-221 and let-7a). Another study identified a miRNAs profile counting only miR-155 and let-7a-2 [121]. The pooled results of a meta-analyse including 28 articles [234] revealed that high expression of miR-125b, miR-21 and miR-200c were negatively associated with survival. Conversely, high expression of miR-124, miR-365, miR-32, miR-148b, miR-146a and miR-375 were significantly associated with better prognosis [234].

However, the critical point with EMT is the associated chemo-resistance [235,236,237,238]. Therefore, understanding the mechanistic of EMT is important to assess treatment strategies for patients with NSCLC.

Upregulation or reactivation of inducing EMT transcription factors activates survival pathways (NF-κB, AKT), proliferation pathways (upregulation of EGFR and MET) and modulates the activity of Bcl-2 family members thereby favouring antiapoptotic signals [25]. Resistance to genotoxic agents such as anthracyclines, platinium-based drugs or spindle poison was associated to EMT. Data suggests that oxaliplatin-resistant colorectal cancer [239], gemcitabine-resistant tumour cells [240] tamoxifen-resistant breast cancer [241], gemcitabine-resistant pancreatic cancer, radiotherapy-resistant endometrial carcinoma [242] and radiotherapy-resistant ovarian cancer cells [243] harbour phenotypes of EMT [25]. Similar results were observed in NSCLC cell lines for cisplatin [244], docetaxel in A549 and for the pemetrexed-ciplatin combination [245,246]. Moreover resistance to treatment in adjuvant setting was also reported [247]. Increased viability or drug resistance could be due to lower levels of ROS and subsequent better genome protection in cell undergoing EMT [25,248]. In NSCLC with EGFR mutations or ALK fusions, EMT was also related to resistance to tyrosine-kinase inhibitors (TKI) [249,250]. NSCLC lines expressing E-cadherin showed greater sensitivity to EGFR inhibition. In contrast, NSCLC lines expressing vimentin and/or fibronectin were insensitive to EGFR inhibition [39]. Data suggested that, in some cases, TGFβ activation is sufficient to induce TKI resistance and, in smokers population, SRC activation could trigger EMT and subsequent EGFR-TKI resistance [251]. Furthermore, EMT increases membrane transporters expression (ABC family, P-glycoprotein) leading to drug active efflux [246,252,253]. Finally EMT-induced decrease of ceramide was associated to chemo-resistance [254,255,256].

The association between EMT and stem-like phenotype in NSCLC cells was shown in several in vitro studies [257,258,259] but the data on this phenomenon in lung cancer patient samples are limited. Koren et al. showed that BMI1 and CD133 (cancer stem-cell markers) were coexpressed in a series of operated NSCLC, giving in vivo evidence of connection between EMT and cancer stem cells [260]. Such tumour subpopulation plays a role in drug resistant tumour cells with abilities of self-renewal, cancer initiation, and further maintenance of tumours [261].

MicroRNAs have been implicated in chemo-resistance as well as in chemo-sensitivity. As seen in the first parts of this review, miRNAs regulate pathways implicated in cell fate: proliferation, apoptosis and differentiation and thereby influence chemotherapy and radiotherapy responses. There are as many models in the literature as different contexts or situations describing a miRNA or a set of miRNAs related to treatment failure. Acquired chemo-resistance, to cisplatin, docetaxel and erlotinib in NSCLC [135,262] was related to up or downregulation of miRNAs linked to EMT : miR-140, miR-628, miR-135b, miR-200b/200c/141, miR-205, miR-197, miR-224, miR-34c, miR301a, miR-636, miR-518f [263]. In *EGFR* mutated tumours CpG island hypermethylation was shown to downregulated miR-200 family members in cells with gefitinib resistance and EMT features. More precisely, miR-200c downregulation was linked to the upregulation of LIN28B a protein favouring stem cell self-renewal [264]. Moreover several afatinib-resistant NSCLC cell lines also displayed EMT features and epigenetic silencing of miR-200c suggesting that miR-200 downregulation could be a common event in TKI acquired resistance [252].

Furthermore, an opposite point-of-view must be presented: some observations lead to consider the epithelial phenotype as drug-resistant. Byers et al. proposed a 76-gene EMT signature to classify NSCLC cell lines into distinct epithelial and mesenchymal groups [265]. Surprisingly, *EGFR*-mutated cell lines H1975 and H820, carrying the acquired-resistance mutation T790M, were classified as epithelial. Furthermore, they found a trend towards greater relative sensitivity in mesenchymal cells as compared to epithelial for cisplatin, gemcitabine and vinorelbine. This observation was also supported by the the work of Miow et al., who reported that ovarian cancer cell lines with an epithelial pattern were more resistant to cisplatin than those with a mesenchymal pattern [266]. This concept remains poorly documented and is largely counterbalanced by abundant literature on mesenchymal status-driven drug resistance. However it may be related with two distinct models of EMT: acquired phenotype of EMT following drug, growth factor or EMT-transcription factor treatment versus inherent phenotype of EMT related to the nature of the cancer cell [1,265,266].

Drug resistance and EMT have been clearly associated in different situations and genetic backgrounds but the mechanisms underlying drug resistance remain under investigation however some explanations could rely on miRNAs expression. 

## 6. Therapies Targeting EMT

Inhibition of EMT could restore senescence and apoptosis capacity [46]. In a recent review, Malek et al. proposed 3 distinct strategies to target EMT through (a) extracellular inducers of EMT, (b) EMT-transcription factors and (c) downstream effectors of EMT inhibition [267]. 

First (a), inhibition of extracellular EMT-inducer pathways could rely on TGF-β blockade using rapamycin, 17-AAG [268], SB-431542 [269] or TGF-β receptor specific inhibitors such as EW-7195, EW-7203 and EW-7197 [270,271,272,273]. Only LY2157299 an oral TGF-βRI tyrosine kinase inhibitor reached clinical trials and is currently tested in several cancers, excluding NSCLC despite previous encouraging preclinical results [274]. Direct blockade of TGFβ using human TGFβ-antibody (fresolimumab) was not yet explored in NSCLC. Besides TGFβ blockade, interesting results were shown using LDN57444, a specific small molecule inhibitor, targeting ubiquitin carboxy-terminal hydrolase L1 (UCH-L1). LDN57444 was shown to downregulate HIF-1α, suppress EMT features and reduce the incidence of distant metastases [275,276]. Concerning extracellular EMT inducers, MMP as metastases-related cellular enzymes could appear as good candidates. However, MMP inhibitors failed in clinical trials likely due to the complexity of the metastatic process [277].

Second (b), inhibition of EMT transcription factors was analysed as a therapeutic option and illustrated by results based on TWIST1 [41,42,45,278,279,280]; PRMT1 [281] and SNAIL family and ZEB1/2 inhibition [20,282]. EMT-transcription factors are challenging therapeutic targets due to their heterogeneous expression and to the complexity of the EMT regulation network [283,284]. However, several agents were proved efficient in cell lines, including plant extracts: moscatilin [285], fucoidan [286], quercetin [287], thymoquinone [288], imipramine blue [289] and finally, in vitro and in vivo data on NSCLC suggest a potential impact of the harmala alkaloids to downregulate TWIST1 [267]. Targeting glycosylation pathways that regulate EMT transcription factors should be an interesting mean of treatment. Aberrant glycosylation was associated with carcinogenesis and aggressiveness in many cancers including NSCLC [290,291] and inhibitors of one of the main glycosylation enzyme are in preclinical pipelines [292,293]. Moreover chromatin modulators such as drugs targeting histone methyltransferases (G9a and EZH2) and demethylases which contributes to EMT as E-cadherin repressors are promising epigenetic oncotargets [294,295]. Finally, histone deacetylation can be inhibited by HDAC inhibitors (butyrate, trichostatin A and Suberoylanilide hydroxamic acid which is the FDA-approved Vorinostat) and have been shown in preclinical studies to selectively target cancer cells by inducing apoptosis, cell cycle arrest, suppression of tumour angiogenesis, metastasis and invasion at least partially through upregulating E-cadherin [267,296,297]. 

Third (c), targeting the downstream effectors of EMT such as E-cadherin, N-cadherin, vimentin and HoxA9 may offer some possibilities [267].

MicroRNAs have emerged as a class of therapeutics targets. However there are some limitations due to the fact that miRNAs have typically many targets. This can either be harmful as it limits specificity but can also be interesting to consider if miRNAs blockage leads to the inhibition at different levels of a pathway, using a single agent. Therapeutic strategy could be either direct administration of anti-miRNA (antisens miRNA) to block OncomiR, or restoration of miRNAs expression to reactivate miRNAs with onco-suppressive functions [206,298,299,300]. Local delivery of miR-200 members into the tumour endothelium showed reduction of metastasis and angiogenesis in several experimental models of ovarian, lung, renal and basal-like breast cancers [301]. In NSCLC, enforced expression of miR-145 inhibited EMT and metastatic ability [208].

Nevertheless, inhibiting or reversing EMT could also lead to a serious adverse event: favouring MET and thus colonisation of metastatic sites by circulating tumour cells [67,302]. Moreover, the intra-tumour and inter-tumour heterogeneity in case of multiple sites, and the dynamic nature of epithelial plasticity in cancer suggests that to be efficient the strategy should be multimodal and not focusing only on a single EMT-related target. As examples, combination of chemotherapy and anti-miRNA strategies and also models of miRNA replacement therapy were tested in vitro and in mouse models [264,303]. Sato et al. showed that the introduction of miR-200c using pre-miR-200c caused LIN28B suppression in cells with acquired EGFR-TKI resistance that harboured EMT features (HCC4006 after chronic exposure to gefinitib) [264]. Van Roosbroeck et al. proposed the use of cisplatin and anti-miR-155 in a mouse model of athymic nude mice (intrapulmonary injections of A549 cells stably infected with lentivirus containing a miR-155-overexpressing lentiviral vector) with significant results in term of primary tumour size and mediastinal lymph nodes [303].

Finally EMT is pointed out as a mechanism of resistance to immunotherapy and is involved in the shaping of the immune microenvironment. It was recently shown in NSCLC that tumours with EMT features expressed PDL1 and other immune checkpoints molecules and suggested that EMT should be further investigated as a predictor of response to immunotherapies [304]. Immunotherapy should then be considered as an anti-EMT therapy [305,306,307].

## 7. Conclusions

EMT is a highly regulated multistep process that is implicated in cancer progression through activation of proliferation pathways, loss of response to apoptotic signals, gain of stem cell properties, matrix remodelling and mobility. EMT involves various signalling pathways and crosstalk as well as a network of transcription factors. Upstream non-coding RNAs such as miRNAs have emerged as potent modulators of EMT. The physiopathology of the EMT process is highly dependent upon the cellular model, the environment and the EMT stimulating factors. Therefore cells undergoing EMT may express different markers. Moreover quantifying the degree of EMT in a tumour remains a challenging task due to its transient and reversible nature. However, some features such as loss of E-cadherin, reactivation of TWIST1, ZEB1 and SNAIL and downregulation of the miR-200 cluster could be common features of EMT in NSCLC.

Because of its link with metastasis and resistance to treatment, EMT has emerged as a useful prognosis and predictive marker but there is yet no clinical application in NSCLC. Our understanding of EMT is growing and may enable us to forward EMT characterization to the clinics. The development of methods to investigate molecular profiles or EMT signature using small amounts of tissues and FFPE sample will help validate EMT markers in clinical settings. Finally, EMT is a promising therapeutic target to overcome drug resistance in NSCLC in patients treated with chemotherapy, targeted therapies and immunotherapies. Because of the complexity of the EMT regulation network, the treatment will rely on integrative personalize care and high throughput molecular screenings.

## Figures and Tables

**Table 1:** List of EMT involved miRNAs based on NSCLC cell lines studies, with details on the involved pathways (green, promote EMT; red, suppress EMT; blue, controversial).

MicroRNAs	Pathway	Cell Lines	References
miR-10a	XRN2	H441, A549, HOP62	[134]
miR-15b	PEBP4	A549	[135]
miR-17	TGF-β	A549	[136]
miR-23a	E-cadherin	A549	[137]
miR-26a	EZH2	SPC-A1, H1299	[138]
miR-30a	SNAIL	A549, Calu1/3, H1299, H1395	[139,140]
miR-34a	NOTCH1	H1299, H460	[141]
miR-129*	MCRS1	801D, SPC-A1, GLC-82, EPLC-32M1, A549, H292, 16HBE, PT67	[142]
miR-134	ITGB1, MAGI2, FOXM1	A549, H1299, A549, LC2/ad, PC3, PC9, RERF-LCKJ, RERF-LCMS, PC14, ABC-1	[143,144,145]
miR-138	GIT1, SEMA4C	A549, 95D, H23	[146,147]
miR-145a-5p	TWIST1	H1299	[148]
miR-149	FOXM1	A549	[149]
miR-151-5p	TWIST1	H1299	[148]
miR-154	ZEB2	A549	[150]
miR-155	ZEB1	HCC827	[151]
miR-206	PI3K/AKT	A549, 95D	[152]
miR-221	na	H460, H838, H1299, H3255, HCC4006, HCC4011	[153]
miR-222	na	H460, H838, H1299, H3255, HCC4006, HCC4011	[153]
miR-337-3p	TWIST1	H1299	[148]
miR-374a	Axl	HCC827, Calu1	[154]
miR-452	BMI1	A549, H460	[155]
miR-483-5p	ALCAM	A549, PC9	[156]
miR-487b	MAGI2	A549	[144]
miR-520f	ADAM9	A549	[157]
miR-543-3p	TWIST1	H1299	[148,158]
miR-544a	Cadherina 1	95C, 95D	[159]
miR-548b	Axl	HCC827, Calu1	[154]
miR-655	MAGI2	A549	[144]
miR-1271	FOXK2	A549, H520, H1299, H358, H460	[160]
miR-1246	Stem cells	A549, HCC1588	[161]
miR-1290	Stem cells	A549, HCC1588	[161]
let-7	HMG2A	H1975, H1299, H1650	[162]
let-7c	Hedgehog	A549, H1299	[163]

**Table 2:** List of EMT involved miRNAs based on NSCLC tissue samples and patients’ series studies, with details on the involved pathways and the clinical impact (green, promote EMT; red, suppress EMT) (TKI, Tyrosine-Kinase Inhibitor).

MicroRNAs	Pathways	Clinical Impact	References
miR-16	HDGF	Cell growth and motility	[164]
miR-21	STAT3, IL-6	Carcinogenesis	[165]
miR-29b	SPARC	Cancer progression	[166]
miR-30	MMP19	Metastases	[167]
miR-30c	E-cadherin, vimentin, SNAIL	Invasion	[168]
miR-31	ERK1/2	Lymph spread, survival	[169]
miR-33a	TWIST1	Metastases	[170]
miR-92b	RECK	Cell growth and motility	[171]
miR-96	Foxf2	Invasion and metastases	[172]
miR-124	CDH2, ZEB1	Cell migration and invasion	[173,174]
miR-127	feed-forward regulatory loop	TKI resistance	[175]
miR-132	ZEB2	Cell migration and invasion	[176]
miR-135a	KLF8	Cell migration and invasion	[177]
miR-135b	Hippo signaling pathway	Metastases	[178]
miR-143	CD44	Cell migration and invasion	[179]
miR-145	SMAD3	Invasion	[180]
miR-146a	IRS2	Cancer progression	[181]
miR-148a	ROCK1	Lymph spread	[182]
miR-150	p53	Cell proliferation	[183]
miR-182	Foxf2	Invasion and metastases	[172]
miR-183	Foxf2	Invasion and metastases	[172]
miR-184	c-Myc	Overall and disease-free survival	[184]
miR-193a	WT1-E-cadherin axis + ERBB4/PIK3R3/mTOR/S6K2 signaling pathway	Metastases	[185,186]
miR-196a	HOXA5	Cell proliferation, invasion	[187]
miR-196b	NF-κB, Homeobox A9	Cell invasion and migration	[188]
miR-200	ZEB1, Foxf2	Invasion and metastases	[172,189]
miR-200c	E-cadherin, ETAR, NFkB	Invasion and metastases	[190,191,192,193]
miR-203	SMAD3	Invasion	[180]
miR-205	CRIPTO1	TKI resistance	[194]
miR-214	Sufu	Metastases	[195]
miR-338-3p	Sox4	Metastases	[196]
miR-361	FOXM1	Cell proliferation and invasion	[197]
miR-375	YAP1	Neuroendocrine features	[198]
miR-451	RAB14	pTNM, Lymph spread	[199]
miR-489	SUZ12	Cell invasion	[200]
miR-490	poly r(C)-binding protein 1	Metastases, lymph spread	[201]
miR-589	HDAC5	Cell migration and invasion	[202]
miR-541	TGIF2	Cancer progression	[203]
miR-638	SOX2	Cell proliferation and invasion	[204]

## Supplementary Materials

The following are available online at http://www.mdpi.com/2072-6694/9/8/101/s1. Table S1: MicroRNA signatures in NSCLC [120,121,122,123,124,125].
